# A Pathway to Chordoma Treatment: A Review on CDKN2A and Therapeutic Targeting

**DOI:** 10.3390/cancers18162688

**Published:** 2026-08-19

**Authors:** Muneeb Mohiuddin, Benjamin Vaca, Elijah Haynal, Othman Bin-Alamer, Peter Zaki, Hussam Abou-Al-Shaar, Georgios A. Zenonos, Hector A. Perez, Miguel Lopez-Gonzalez, Zachary C. Gersey

**Affiliations:** 1 Department of Neurosurgery, University of Miami, Miami, FL 33136, USA; 2 Department of Neurosurgery, Loma Linda University, Loma Linda, CA 92354, USA; ehaynal@students.llu.edu (E.H.); mlopezgonzalez@llu.edu (M.L.-G.); 3 Department of Neurosurgery, University of Utah, Salt Lake City, UT 84132, USA; 4 Department of Neurosurgery, McGovern Medical School, Houston, TX 77030, USA; 5 Department of Neurosurgery, University of Pittsburgh Medical Center, Pittsburgh, PA 15213, USA; 6 Department of Otolaryngology-Head and Neck Surgery, Loma Linda University, Loma Linda, CA 92350, USA

**Keywords:** chordoma, CDKN2A, skull base, CDK inhibitors, cancer genetics

## Abstract

**Background:** Chordoma is a rare malignant bone tumor thought to arise from remnants of the embryonic notochord. Its management remains challenging because of its proximity to critical neurovascular structures and its high propensity for local recurrence. Current standard treatment consists of maximal safe resection followed by radiotherapy, yet durable disease control remains difficult to achieve in many patients. Emerging evidence suggests that cyclin-dependent kinase inhibitor 2A (CDKN2A) loss is a recurrent molecular event in chordoma and may serve as both a prognostic biomarker and a therapeutic target. **Methods:** A literature search was performed by acquiring articles containing “(CDKN2A or p16) AND (chordoma or notochordal tumor)”, and “chordoma 9p21”. Of the 41 articles retrieved, 17 met the inclusion criteria. **Results:** Homozygous and heterozygous **CDKN2A** deletions were frequently identified in chordoma using fluorescence in situ hybridization and genomic sequencing approaches. These alterations were commonly associated with loss of p16^INK4A expression, the protein product of **CDKN2A**, supporting a role in tumorigenesis and disease progression. Epigenetic mechanisms may also contribute to reduced p16^INK4A expression in a subset of tumors. Preclinical studies in **CDKN2A**-deficient chordoma cell lines and patient-derived xenografts demonstrated sensitivity to cyclin-dependent kinase 4 and 6 (**CDK4/6**) inhibitors, including palbociclib, flavopiridol, and abemaciclib. Combination strategies pairing palbociclib with buparlisib or rapamycin produced greater antitumor effects, particularly in p16^INK4A- and PTEN-deficient models. In addition, CDKN2A loss has been associated with adverse clinical features in selected cohorts, although its independent prognostic significance remains unclear. Available preclinical evidence further suggests that chordomas lacking p16^INK4A expression and retaining retinoblastoma pathway dependence may be particularly susceptible to **CDK4/6** inhibition. **Conclusions:** CDKN2A loss is a recurrent molecular alteration in chordoma that is associated with reduced p16^INK4A expression, adverse clinicopathologic features, and less favorable outcomes in selected cohorts, although its independent prognostic significance remains inconsistent. Preclinical data support CDK4/6 inhibition, particularly in biomarker-selected CDKN2A-deficient tumors, and suggest that combination approaches targeting complementary pathways such as PI3K/mTOR may further enhance therapeutic efficacy. Together, these findings support the clinical relevance of CDKN2A as both a prognostic biomarker and a promising therapeutic target in chordoma.

## Simple Summary

Chordoma is a rare cancer of the spine and skull base that is difficult to treat once it recurs or spreads. One of the most frequent molecular changes in chordoma is loss of the CDKN2A tumor suppressor gene, which can disrupt normal control of cell division and may create a vulnerability to drugs that block cyclin-dependent kinases 4 and 6. This review summarizes current evidence on how often CDKN2A is altered in chordoma, whether these changes are linked to clinical outcomes, and how they may guide targeted treatment. We also examine the limitations of using CDKN2A status alone, the role of related molecular pathways, clinical trial findings, and potential resistance mechanisms. These findings support more precise biomarker-based patient selection and future studies of rational combination therapies.

## 1. Introduction

Chordoma is a rare, malignant bone tumor thought to arise from remnants of the embryonic notochord and primarily affects the axial skeleton, particularly the skull base and sacrum [1]. Although typically slow growing, chordoma is locally aggressive and poses major clinical challenges because of its proximity to critical neurovascular structures. With an estimated incidence of 0.09 per 100,000 persons annually, chordoma accounts for approximately 1–4% of primary bone malignancies [2]. Prognosis remains poor due to high rates of local recurrence and limited effective systemic treatment options. Current standard management consists of maximal safe surgical resection followed by adjuvant radiotherapy; however, durable disease control remains difficult to achieve in many patients, and progression or metastasis can still occur [3]. Given these limitations, there is a strong need to identify molecularly informed therapeutic strategies that may improve outcomes for patients with chordoma.

Cyclin-dependent kinase inhibitor 2A (CDKN2A), located on chromosome 9p21, is a tumor suppressor gene that encodes two distinct proteins, p16^INK4A and p14^ARF, through alternative reading frames. These proteins play essential roles in cell cycle regulation and tumor suppression. p16^INK4A functions as an inhibitor of cyclin-dependent kinases 4 and 6 (CDK4/6), preventing phosphorylation of the retinoblastoma (Rb) protein and restricting G1-S cell-cycle progression [4,5]. In parallel, p14^ARF regulates the p53 pathway by inhibiting mouse double minute 2 homolog (MDM2), thereby stabilizing p53 and promoting cell-cycle arrest or apoptosis in response to oncogenic stress [4,6]. Through these complementary functions, CDKN2A serves as a key regulator of both the Rb and p53 pathways, and its inactivation has been implicated in the pathogenesis of many cancers, including chordoma [5].

Among the mechanisms of CDKN2A inactivation, homozygous deletion is one of the best characterized and has been associated with tumorigenesis across multiple malignancies [5,7]. Loss of p16^INK4A promotes unchecked CDK4/6 activity, excessive Rb phosphorylation, and unregulated cell cycle progression, whereas loss of p14^ARF impairs p53-mediated apoptotic signaling [5,8,9,10]. Together, these alterations may contribute to increased proliferation and have been associated with adverse clinicopathologic features and less favorable outcomes in selected cohorts. In chordoma, emerging evidence suggests that CDKN2A loss is a recurrent molecular event with potential biological and clinical relevance.

This review aims to provide clinicians and scientists with a comprehensive understanding of CDKN2A’s role in chordoma tumorigenesis and to highlight therapeutic strategies targeting CDKN2A-associated pathways. We focus on the frequency and mechanisms of CDKN2A alteration, its prognostic significance, and the rationale for CDK4/6-directed therapies in chordoma.

## 2. Methods

**Figure 1:**
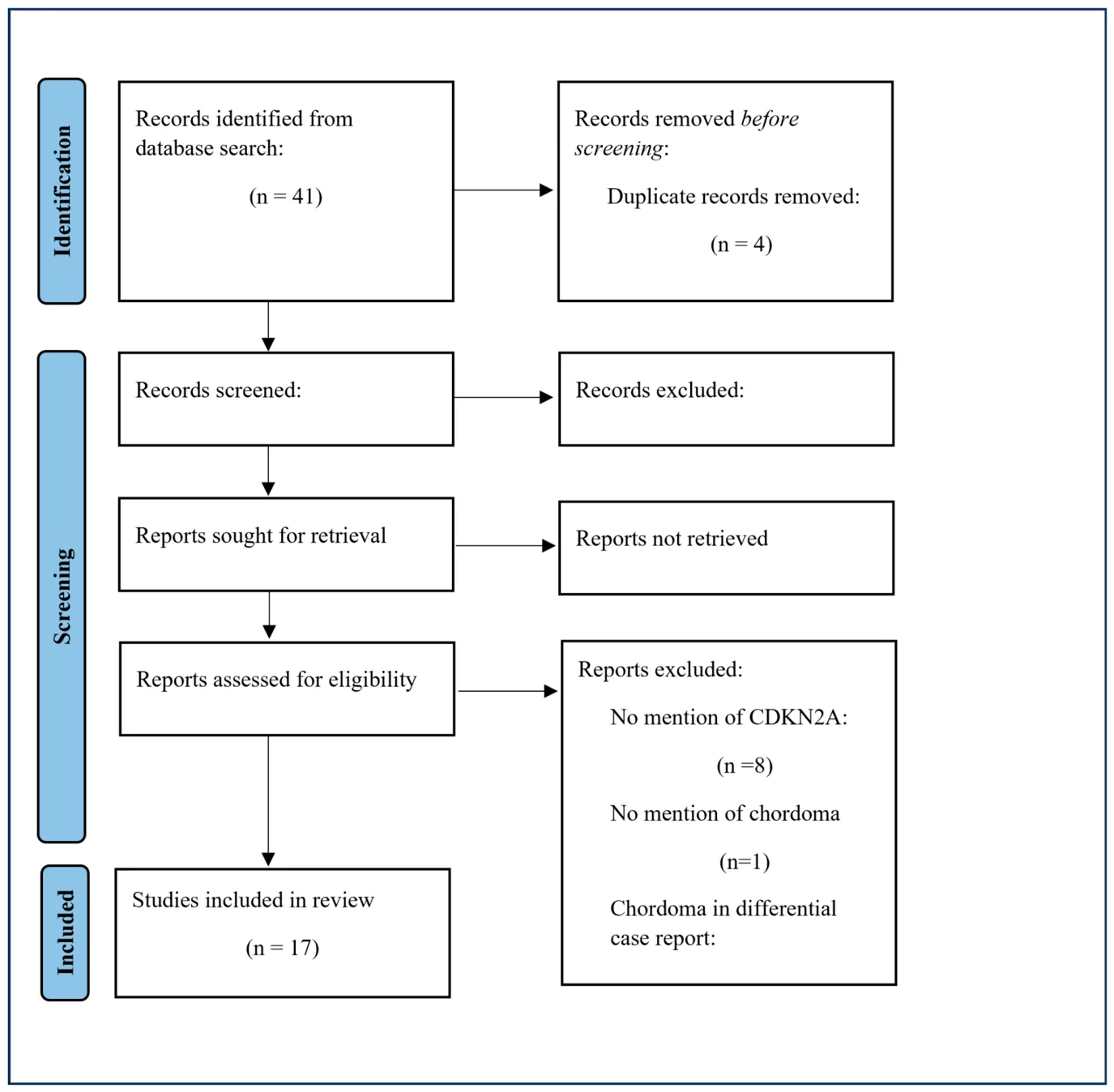
Literature identification and study selection process for the structured narrative review.

This study was conducted as a structured narrative review supported by a predefined PubMed search and a transparent study-selection process. Using the Medical Subject Heading (MeSH) database system of PubMed, we performed a literature search between the years 1998 and 2025 for all articles containing the terms “(CDKN2A or p16^INK4A) AND (chordoma or notochordal tumor)”, and “chordoma 9p21”: articles were not limited to humans. Case reports and reviews were excluded. A total of 41 articles were found during the initial investigation, of those 4 were duplicates, leading to a total of 37 articles to review. Studies were eligible if they reported CDKN2A/9p21 or p16^INK4A status in chordoma, examined its prognostic or biological significance, or evaluated therapeutic targeting in molecularly characterized chordoma models or patients. A total of 17 were selected to be investigated. 20 were excluded for not reporting CDKN2A status or lacking data on therapeutic targeting of CDKN2A-altered tumors. The results of our literature screening are summarized in Figure 1.

To broaden the therapeutic scope beyond CDK4/6 inhibition, a supplementary targeted PubMed search was conducted during revision from 26 July 2026–6 August 2026. “Chordoma” was combined with terms related to EGFR/mTOR, TBXT/brachyury, PDGFR, MTAP-associated vulnerabilities, SMARCB1/EZH2, and immunotherapeutic or antiangiogenic strategies, with CDKN2A and p16^INK4A included where relevant. Primary preclinical and clinical studies of molecular therapies were eligible, whereas reviews, case reports, and studies focused exclusively on surgery or radiotherapy were excluded. Studies not stratified by CDKN2A or p16^INK4A status were included only for broader therapeutic context and were not interpreted as evidence of CDKN2A-specific efficacy. ClinicalTrials.gov was additionally searched during revision to identify ongoing or unpublished interventional studies relevant to CDKN2A/p16^INK4A or related molecular therapies.

Because this was a structured narrative review encompassing substantial heterogeneous genomic, prognostic, preclinical, and clinical studies, no formal risk-of-bias instrument or quantitative study quality score was applied. Studies were instead critically interpreted according to design, cohort, or model size, anatomical tumor location, and assay methodology.

## 3. Results

**Figure 2:**
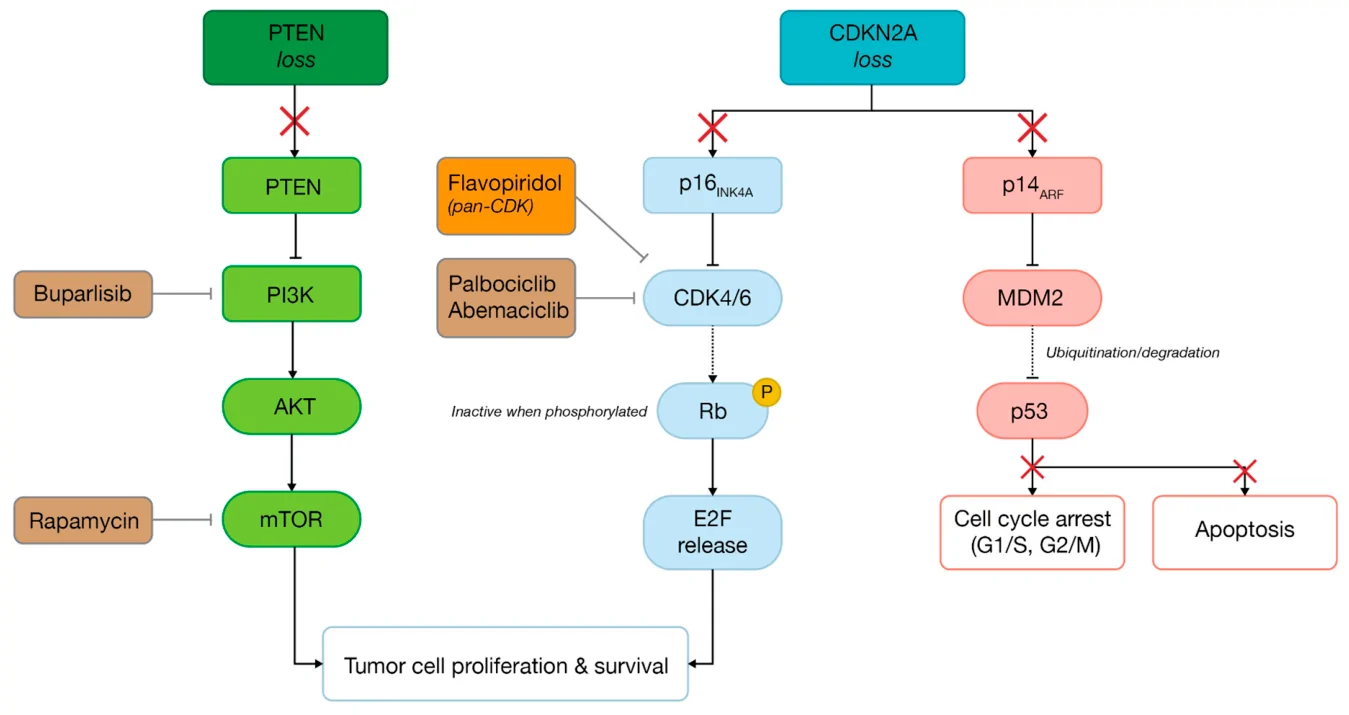
Mechanistic consequences of CDKN2A inactivation in chordoma. CDKN2A encodes two tumor suppressors, p16^INK4A and p14^ARF, which regulate the CDK4/6–Rb–E2F pathway and the MDM2–p53 axis, respectively. Loss of p16^INK4A leads to unchecked CDK4/6 activity, Rb phosphorylation, E2F release, and G1→S cell-cycle progression. Loss of p14^ARF permits MDM2-mediated p53 degradation, impairing p53-driven cell-cycle arrest and apoptosis. In parallel, loss of PTEN, a phosphatase that normally restricts PI3K signaling, results in disinhibition of the PI3K/AKT/mTOR axis, providing a complementary proliferative and pro-survival stimulus. Selective CDK4/6 inhibitors (palbociclib, abemaciclib) and the pan-CDK inhibitor flavopiridol act upstream to prevent Rb phosphorylation and restrain proliferation, while PI3K (buparlisib) and mTOR (rapamycin) inhibitors target the parallel axis.

Deletion of CDKN2A at chromosome 9p21.3 is among the most recurrent genomic alterations reported in chordoma. Across the studies included in this review, CDKN2A status was evaluated using a range of molecular and genomic approaches, including comparative genomic hybridization (CGH), single nucleotide polymorphism (SNP) arrays, whole-genome (WGS) or whole-exome sequencing (WES), fluorescent in situ hybridization (FISH), methylation profiling, and immunohistochemistry (IHC). Collectively, these studies suggest that CDKN2A/B loss is frequent, varies by anatomical site, and is associated with reduced p16^INK4A expression, more aggressive tumor biology, and poorer clinical outcomes (Table 1). They also provide insight into alternative mechanisms of p16^INK4A loss and support the therapeutic rationale for CDK4/6 inhibition in selected chordoma models (Figure 2).

### 3.1. Frequency of CDKN2A/B (9p21.3) Deletions in Chordoma

In an early genome-wide copy number analysis of 20 sporadic chordomas, including two clival tumors and predominantly spinal/sacral lesions, Le et al. identified copy number loss at 9p21.3 in 16 of 20 tumors (80%), including homozygous deletions in 6 cases (30%) [11]. Most patients in this cohort (15/20) had received prior radiation therapy, which may have influenced the observed genomic landscape.

In one of the largest studies to date, Cottone et al. evaluated CDKN2A alterations and p16^INK4A protein expression using FISH and IHC across 384 chordoma specimens from 320 patients [12]. CDKN2A copy number alterations were identified in 61% of informative cases (167/274), with copy number loss representing the predominant event (83% of altered cases). Importantly, homozygous deletion of CDKN2A was consistently associated with complete loss of p16^INK4A protein expression. Similarly, Passeri et al. performed whole-exome sequencing on 64 chordoma tumors and found homozygous deletion of CDKN2A/B to be the most frequent copy number alteration, present in 12 of 60 evaluable samples (20%) [13].

In skull base chordomas specifically, lower frequencies of deletion have generally been reported. Diaz et al. used high-resolution SNP microarray analysis and identified focal CDKN2A/B deletions in 4 of 18 primary clival tumors (22%), a markedly lower frequency than that observed in sacral chordomas [14]. Huo et al. likewise reported deletions in only 3 of 46 tumors (6.5%) in their internal skull base cohort, although this increased to 16 of 46 tumors (34.8%) in an external validation cohort [15]. Using WGS in 80 skull base chordomas, Bai et al. identified CDKN2A alterations in 11 cases (13.8%), including 9 homozygous deletions [16]. Baluszek et al. further observed enrichment of CDKN2A/B loss within one of two methylation-defined clusters, with 9 of 10 tumors in that cluster demonstrating loss, although the specific type of deletion and its relationship to survival were not reported [17].

In an analysis of 99 chordoma samples, Hsia et al. found loss of heterozygosity involving CDKN2A in 27 cases (27.3%), making it the most common copy number alteration in the cohort [18]. CDKN2B co-deletion was nearly as frequent, occurring in 25 of 99 samples (25.3%), consistent with the close genomic proximity of these genes. Similarly, Chan et al. analyzed 86 chordoma tumor samples using targeted next-generation sequencing in a multicenter cohort and found CDKN2A/B to be the most frequently altered locus, present in 28 patients (32.6%). All documented CDKN2A/B alterations were deletions rather than single-nucleotide variants or amplifications. Progressive disease was more common among patients with CDKN2A/B alterations than in the overall cohort (58.8% vs. 46.3%), although progression data were available for only 67 patients [19].

**Table 1:** Overview of major genomic and molecular studies assessing CDKN2A/B (9p21) alterations in chordoma. The table summarizes cohort size, tumor location, analytic methodology (e.g., FISH, IHC, CGH, SNP microarray, WGS/WES), and principal findings related to CDKN2A/B loss, p16^INK4A expression, associated genetic alterations, and proposed mechanisms of inactivation.

Study (Year)	Cohort/Sample Details	Methodology	Key Genetic Findings	Independent Association with Outcomes?
Sommer et al. (2010) [20]	30 chordoma specimens (14 skull-base tumors)	IHC analysis (CDKN2A and MTAP)	• Absent CDKN2A expression observed in 74% of samples • Loss of MTAP expression in 37% of cases, which always occurred alongside CDKN2A loss	No; association did not remain significant in multivariable analysis
Le et al. (2011) [11]	21 chordoma samples from 20 patients (including 2 clival; many had prior radiation)	Array Comparative Genomic Hybridization (CGH) and IHC	• CDKN2A/B loss detected in 16/20 patients (6 with homozygous deletions) • Both skull-base samples showed homozygous deletion • Some cases exhibited deficient p16^INK4A expression despite an intact CDKN2A gene • A single case of promoter methylation was noted	Not assessed
Diaz et al. (2012) [14]	22 chordoma samples (21 from the clivus)	SNP Microarray	• Focal chromosome 9 deletions, including CDKN2A/B loss, were observed in 4/18 primary clival chordomas • Aneuploidy of chromosome 3 • Epigenetic regulation noted for FHIT, with minimal/absent expression in 67% of tumors	Not assessed
Zenonos et al. (2018/2019) [21]	105 skull-base chordoma samples	Fluorescence In Situ Hybridization (FISH)	• Detection of homozygous deletions at the 9p21 (and 1p36) loci	Yes, homozygous 9p21 deletion independently predicted shorter progression-free survival after surgery and radiotherapy in multivariable Cox analysis.
Cottone et al. (2020) [12]	384 chordoma specimens (including 90 skull-base tumors)	FISH and Immunohistochemistry (IHC)	• 61% of samples exhibited CDKN2A copy number alterations (83% of these were copy number losses) • Homozygous deletion of CDKN2A resulted in 100% loss of its protein product p16^INK4A • Some cases showed p16^INK4A loss despite an intact CDKN2A gene, suggesting additional regulatory mechanisms	Not assessed
Bai et al. (2021) [16]	80 skull-base chordoma patients (predominantly East Asian)	Whole Genome Sequencing (WGS)	• Low overall mutational burden • Rare TBXT duplication (detected in only 2 cases) • CDKN2A alterations in 11 patients (9 with homozygous deletion) • Other potential drivers: PBRM1+, SETD2+, TBXT/LYST+ • Notable arm-level 9q deletion findings	No, CDKN2A/B alterations were not independently associated with chordoma-specific or recurrence-free survival.
Huo et al. (2022) [15]	46 skull-base chordoma patients; plus an external cohort of 46	Methylation profiling and Copy Number (CN) analysis	• Unsupervised clustering identified two distinct methylation clusters • CDKN2A/2B deletions occurred exclusively in the high-risk cluster • Three methylation sites (cg15645309, cg01234517, cg10847094) were linked to risk (with cg01234517 correlating with higher risk)	Not established for CDKN2A/B alone
Baluszek et al. (2023) [17]	32 skull-base chordomas	DNA Methylation and RNA Sequencing	• Two distinct aberrant methylation clusters were identified • Cluster C had a high proportion of CDKN2A/B losses (9/10 tumors) compared to Cluster I (1/23 tumors)	No
Passeri et al. (2023) [13]	64 chordoma samples (37 skull-base; remaining from spinal locations)	Whole Exome Sequencing (WES)	• CDKN2A was the most frequent copy number variation (observed in 12/60 samples) • Higher prevalence of CDKN2A loss in spinal chordomas compared to skull base • Recurrent tumors showed acquisition of homozygous deletion of CDKN2A/B	No; only PIK3CA retained significance in multivariable analysis
Hsia et al. (2025) [18]	99 chordoma samples from the AACR Project GENIE database	Targeted next-generation sequencing (NGS); copy number variation analysis; tumor mutational burden (TMB) assessment	• Loss of heterozygosity (LOH) involving CDKN2A identified in 27/99 samples (27.3%), the most common copy number alteration in the cohort • CDKN2B co-deletion nearly as frequent, present in 25/99 samples (25.3%) • Pediatric cases demonstrated distinct mutation profiles compared to adults	Not assessed
Chan et al. (2025) [19]	86 chordoma tumor samples from a multicenter cohort	Targeted next-generation sequencing (NGS)	• CDKN2A/B alteration was the most frequently mutated locus, present in 28/86 samples (32.6%); all documented alterations were deletions (no SNVs or amplifications) • By histologic subtype: CDKN2A/B mutation most common in conventional chordoma (24/70, 34%) and chondroid chordoma (3/9, 33%) • Disease progression more frequent in tumors with CDKN2A/B alterations vs. overall cohort (58.8% vs. 46.3%)	Not established; progression data available for 67 patients

### 3.2. Anatomical and Demographic Correlates of CDKN2A Deletion

Le et al. observed homozygous deletions in both skull base tumors in their cohort, whereas Diaz et al. and Passeri et al. reported lower deletion frequencies in skull base chordomas than in sacral or spinal tumors [11,13,14]. These findings support anatomical site as a potential determinant of CDKN2A alteration frequency and further suggest that deletion frequency varies by tumor location.

### 3.3. Mechanisms of CDKN2A Inactivation Beyond Homozygous Deletion

**Figure 3:**
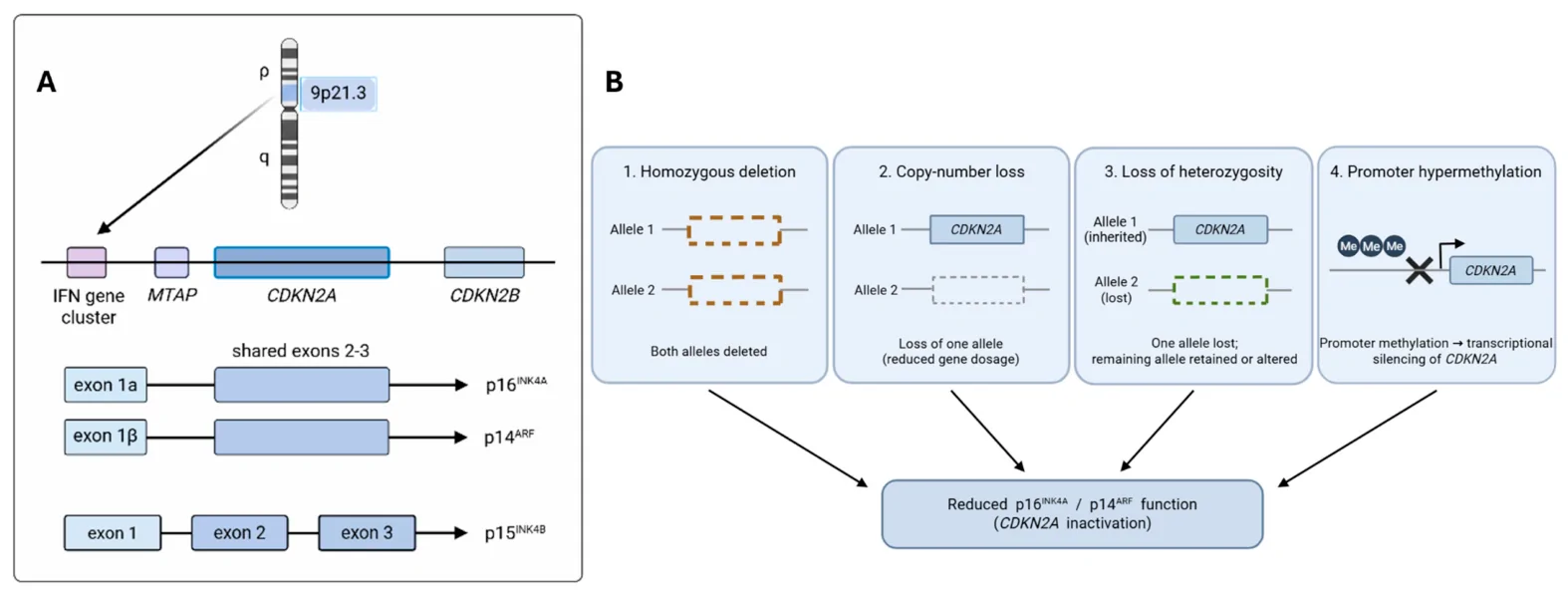
CDKN2A/CDKN2B locus organization and mechanisms of CDKN2A inactivation. (**A**) Schematic representation of the chromosome 9p21.3 locus and exon organization of CDKN2A and CDKN2B. Alternative first exons of CDKN2A generate p16^INK4A and p14^ARF through shared exons 2–3, whereas CDKN2B encodes p15^INK4B. (**B**) Principal mechanisms contributing to CDKN2A inactivation, including homozygous deletion, copy-number loss, loss of heterozygosity, and promoter hypermethylation.

Several studies have explored mechanisms of CDKN2A inactivation beyond genomic deletion (Figure 3). Cottone et al. observed that only 33% of p16^INK4A-negative tumors harbored homozygous CDKN2A deletions, suggesting that additional mechanisms contribute to loss of p16^INK4A expression [12]. In the same study, promoter methylation was found to be rare and insufficient to explain most cases of p16^INK4A loss. Similarly, Le et al. reported that only one of two tumors with loss of p16^INK4A expression despite intact CDKN2A copy number demonstrated promoter methylation, further supporting a limited role for this epigenetic mechanism [11].

In addition to epigenetic silencing, sequence-level alteration may also contribute to CDKN2A inactivation. Prior genotyping studies identified an R58 point mutation in CDKN2A within a region of chromosomal copy loss, suggesting that focal mutation may represent another mechanism of functional inactivation in chordoma [7]. Post-transcriptional regulation has also been proposed as a potential contributor. Cottone et al. evaluated the rs11515 SNP, which was predicted to alter the miR-601 binding site within the 3′ untranslated region of CDKN2A, but found no association with either p16^INK4A protein expression or copy number status [12]. Although this specific mechanism was not supported, other post-transcriptional or translational regulatory processes may still contribute to reduced p16^INK4A expression. This interpretation is further supported by observations that p16^INK4A loss can occur in tumors with disomy, monosomy, heterozygous loss, or copy-number gain at the CDKN2A locus, implying that genomic status alone does not fully explain loss of protein expression.

Beyond homozygous deletion, loss of heterozygosity (LOH) represents a distinct mechanism of CDKN2A inactivation. Under the foundational two-hit model, deletion or loss of one CDKN2A allele is followed by inactivation of the remaining allele through an independent event such as a point mutation, promoter hypermethylation, or focal deletion. Consequently, there is complete functional loss despite the retention of one copy at the copy number level. In chordoma, allelic loss of 9p21.3 appears common, and in a large registry-based analysis, Hsia et al. identified LOH involving CDKN2A in 27.3% of samples, making it the most frequent copy number alteration in their cohort, with CDKN2B co-deletion detected at a nearly comparable frequency [18]. These findings highlight that CDKN2A inactivation in chordoma is frequently the result of biallelic disruption arising from heterogeneous mechanisms, rather than homozygous deletions alone.

### 3.4. Molecular and Cellular Features of CDKN2A-Altered Chordomas

Using immunohistochemistry, Sommer et al. found loss of p16^INK4A protein expression in 74% (20/27) of analyzed chordoma specimens, which was significantly associated with concurrent MTAP loss (*p* = 0.026) [20]. Although loss of p16^INK4A expression was associated with worse disease-free survival on univariate analysis, this relationship did not remain significant in multivariate modeling.

In skull base chordomas, Zenonos et al. showed that homozygous 9p21 deletions were associated with shorter progression-free survival. Stratification by deletion burden (0–3%, 4–24%, and ≥25%) demonstrated an inverse relationship between 9p21 deletion and progression-free survival, supporting the prognostic relevance of this alteration and informing a molecular risk stratification tool to guide adjuvant radiotherapy decisions [21].

Similarly, Passeri et al. reported that CDKN2A/B loss was associated with higher Ki-67 indices (≥6%) and shorter progression-free survival on univariate analysis, although only PIK3CA mutations retained significance in multivariate models. Notably, in two matched primary-recurrence pairs, homozygous CDKN2A/B deletions were acquired in recurrent tumors, suggesting a potential role in progression and tumor aggressiveness [13].

Huo et al. provided further support for the association between CDKN2A/B loss and aggressive disease by showing that these deletions were confined to the poor-prognosis methylation cluster [15]. Although Baluszek et al. did not identify a direct association between CDKN2A/B status and overall survival, they found that greater overall copy number alteration burden correlated with worse survival, suggesting that CDKN2A/B loss may occur within a broader pattern of genomic instability linked to adverse outcomes [17].

Chan et al. further reported that disease progression was more frequent among tumors harboring CDKN2A/B alterations than in the overall cohort, supporting an association between CDKN2A/B loss and a more aggressive clinical phenotype [19].

### 3.5. Clinical Relevance of CDKN2A Loss: Prognostic and Predictive Implications

Several studies support the clinical relevance of CDKN2A loss in chordoma as both a prognostic marker and a potential predictor of response to targeted therapy. Abdallah et al. evaluated the prognostic significance of 9p21 deletion in skull base chordomas by stratifying 152 tumors into three molecular risk groups based on the extent of 1p36 and 9p21 loss [21]. Group A (low risk) was defined by <15% 1p36 deletion and <4% 9p21 deletion, whereas Group C (high risk) had >15% 1p36 deletion and >25% 9p21 deletion; Group B comprised tumors falling between these thresholds. Progression-free survival differed substantially across groups, with mean progression-free survival of 110 months in Group A, 59 months in Group B, and 16 months in Group C. Adjuvant radiotherapy improved outcomes in Groups B and C but conferred no benefit after gross total resection in Group A tumors, supporting the use of deletion burden as a clinically meaningful prognostic marker [22].

Seeling et al. further evaluated the prognostic effect of p16 and PTEN loss in chordoma. Although loss of either marker alone was not significantly associated with overall survival, the combined loss of both p16 and PTEN was associated with significantly worse outcomes compared with controls. Patients with concomitant p16 and PTEN loss had a median overall survival of 6.2 years, compared with 15 years in the control group, suggesting that CDKN2A inactivation may have greater prognostic significance when considered in combination with other pathway alterations [23].

The predictive relevance of CDKN2A loss for CDK4/6-targeted therapy was explored by von Witzleben et al. In that study, the authors analyzed 43 chordoma tissue samples and generated 8 independent patient-derived chordoma cell lines for functional testing. Across tissue specimens, more than 90% retained phosphorylated retinoblastoma protein (pRb), whereas approximately 75% demonstrated complete loss of p16 expression, consistent with persistent downstream CDK4/6–Rb pathway dependence despite upstream loss of cell-cycle inhibition. In the derived cell lines, CDKN2A loss ranged from 50% to 86% by FISH, and these models’ showed sensitivity to CDK4/6 inhibition. Palbociclib and abemaciclib both reduced cell viability, although the degree of response varied across lines: U-CH2, U-CH3, U-CH6, and U-CH7 showed relatively high sensitivity to palbociclib, with IC50 values of approximately 50–100 nM, whereas U-CH11 was less sensitive, requiring concentrations of 300–400 nM. Together, these findings suggest that loss of p16 expression in the setting of retained Rb pathway activity may define a biologically plausible responder profile for CDK4/6 inhibition in chordoma [24].

### 3.6. Therapeutic Strategies Targeting CDKN2A-Altered Chordoma

Aberrant activation of the CDK4/6 pathway, often associated with loss of CDKN2A/p16^INK4A, has been implicated in chordoma cell-cycle dysregulation and provides a rationale for therapeutic CDK4/6 inhibition [12,24]. This disruption promotes unchecked cell cycle progression through the G1-S checkpoint, thereby enabling sustained tumor cell proliferation (Figure 2). In this section, we provide a comprehensive review of promising therapeutic agents that inhibit the CDK4/6 pathway to function as a therapeutic strategy for treating patients with chordoma; key agents, genetic profiles, and efficacy data are summarized in Table 2.

#### 3.6.1. Palbociclib Efficacy

Considering the prominence of CDKN2A deletion in chordoma cell lines, palbociclib (PD-0332991), a selective CDK4/6 inhibitor, was evaluated for its effects on chordoma cell proliferation [24]. In this study, the authors analyzed 43 chordoma tissue samples for clinicopathologic features and established 8 independent cell lines from patient-derived tumors for in vitro analysis. By FISH analysis, it was shown that each of the cell lines exhibited CDKN2A loss ranging from 50% to 86%. In mRNA analysis, CDK4/6 and Rb are expressed, which serve as the therapeutic target. These cell lines were then treated with increasing concentrations of palbociclib, measured against MCF7 cells and CAL 51 cells, which served as established positive and negative controls, respectively. All cell lines treated with palbociclib resulted in up to an 18.5% reduction in proliferation.

#### 3.6.2. Palbociclib on Patient-Derived Xenografts

Passeri et al. treated three patient-derived chordoma xenografts with the CDK4/6 inhibitor palbociclib, as well as the PLK1 inhibitor volasertib [25]. Two samples harbored homozygous deletions of the CDKN2A/B genes, while the third sample, serving as the control, presented a PBRM1 pathogenic variant (c.599C>G) without CDKN2A/B deletion.

The three xenografts were derived from a sacral primary tumor displaying a homozygous deletion of CDKN2A/B, a skull base tumor previously irradiated and displaying a homozygous deletion of CDKN2A/B, and a skull base primary tumor with a PBRM1 pathogenic variant, lacking CDKN2A/B deletion, respectively.

To determine the efficacy of palbociclib and volasertib as treatments for chordomas, Passeri et al. measured a tumor growth inhibition (TGI) value, which is used to measure the percentage of reduced tumor growth over time. As such, the authors indicated a meaningful biological effect to occur when the TGI was at least 50%. Palbociclib treatment produced differential effects across the models. Tumors with CDKN2A/B deletions exhibited tumor growth inhibition (TGI) values of 58% and 62%, respectively, relative to untreated controls. By contrast, the tumor bearing the PBRM1 mutation showed limited response, with a TGI of 12%. These results highlight a stronger efficacy of palbociclib in xenografts with CDKN2A/B deletions, compared to those without this genetic alteration.

#### 3.6.3. Additional CDK Inhibitors

In a study by Michmerhuizen et al., 2020, the efficacy of the CDK4/6 inhibitor palbociclib and the broad-spectrum CDK inhibitor flavopiridol (targeting CDK1, CDK2, CDK4, and CDK6) was evaluated in two patient-derived chordoma cell lines [26]. The first sample, UM-Chor1, exhibited no CDKN2A alteration, while the second sample, UM-Chor2, harbored a homozygous deletion of CDKN2A, with subsequent alterations in CDK, EGF, and PI3K pathways. Palbociclib demonstrated moderate efficacy in UM-Chor2, achieving an IC50 of 7.4 μM, indicating sensitivity to CDK4/6 inhibition. In contrast, UM-Chor1, which lacked CDKN2A alterations, was less sensitive, with IC50 values exceeding the maximum tested concentration, suggesting a dependency of CDKN2A-deleted cells on the CDK4/6–Rb pathway. Flavopiridol showed significantly greater efficacy in both cell lines, particularly in UM-Chor2, with an IC50 of 0.3 μM. This suggests that broader CDK inhibition may effectively target chordoma cells regardless of CDKN2A status.

To further assess CDK4/6 inhibition in CDKN2A-altered chordoma, von Witzleben et al., 2015 tested palbociclib and abemaciclib across multiple chordoma cell lines [24]. Abemaciclib produced similar reductions in cell viability, supporting the role of CDK4/6 pathway blockade in limiting chordoma growth. Sensitivity to palbociclib varied among the tested lines: U-CH2, U-CH3, U-CH6, and U-CH7 exhibited IC50 values between 50 and 100 nM, indicating high sensitivity, whereas U-CH11 required higher concentrations (IC50 300–400 nM). This variability highlights the molecular heterogeneity of chordomas and suggests that individual tumor profiles may influence response to CDK4/6-targeted therapies.

#### 3.6.4. Synergistic Effects with Buparlisib and Rapamycin

Seeling et al. demonstrated enhanced anti-tumor effects when palbociclib was combined with rapamycin (an mTORC1 inhibitor) in chordoma cell lines with missing p16 function due to the genomic loss of CDKN2A [23]. Rapamycin was used to target a different cellular mechanism, PI3K/AKT/mTOR signaling, that has been frequently reported to be altered in chordoma studies due to copy number loss at the PTEN gene locus on chromosome 10q23.3 [7,27,28,29]. The combination resulted in synergistic reductions in cell viability compared to singular inhibition of palbociclib and rapamycin. Combining palbociclib with the pan-PI3K inhibitor buparlisib did not show synergistic effects, although buparlisib monotherapy was highly effective in reducing cell viability. These findings highlight the potential benefit of simultaneously targeting the CDK4/6 and mTOR pathways in a subset of chordomas characterized by loss of p16 from CDKN2A deletion and PI3K/AKT/mTOR signaling alteration due to copy number loss of PTEN.

In PTEN-positive + p16-negative cell lines, the response to combination therapy was less pronounced, suggesting that intact PTEN may mitigate the effects of CDKN2A loss. These results highlight the importance of considering both p16 and PTEN status when designing combination treatment strategies targeting the CDK4/6-Rb and PI3K/AKT/mTOR pathways.

#### 3.6.5. Potential Mechanisms of Resistance to CDK4/6 Inhibition

Chordoma-specific evidence regarding resistance to CDK4/6 inhibition remains limited. In a single patient with recurrent chordoma characterized by CDKN2A/p16 loss, palbociclib produced an initial partial response followed by progression during the fifth month of treatment. Paired molecular analysis identified marked E2F1 amplification in the post-progression specimen, suggesting that CDK4/6-independent activation of the Cyclin E–CDK2–E2F axis may have contributed to acquired resistance. However, this observation was derived from a single retrospective case and may also reflect clonal evolution or intratumoral heterogeneity rather than a broadly applicable resistance mechanism.

Other potential resistance mechanisms have been defined primarily in non-chordoma malignancies. For example, Wander et al. identified that a loss or functional inactivation of RB1 may eliminate the downstream target required for CDK4/6-inhibitor activity, while amplification of CCNE2 and activation of alternative signaling pathways, such as AKT1, RAS, ERBB2, and FGFR2, in CDK4/6 inhibitor-resistant breast cancers [30]. Furthermore, breast cancer models examined in a study conducted by Herrera-Abreu et al. demonstrated rapid adaptation to CDK4/6 inhibition through persistent G1–S cyclin expression and noncanonical Cyclin D1–CDK2 signaling [31]. Although these findings provide a rationale for assessing RB1, Cyclin E/CDK2, E2F, and PI3K-pathway alterations in chordoma, their relevance has not been prospectively validated CDKN2A-altered chordoma [30,31].

#### 3.6.6. Additional Therapeutic Strategies Beyond CDK4/6 Inhibition

Although several additional molecular pathways have been investigated therapeutically in chordoma, evidence linking their activity specifically to CDKN2A or p16^INK4A status remains limited. The PI3K/AKT/mTOR pathway represents one of the most directly relevant complementary pathways. Michmerhuizen et al. demonstrated preclinical activity of PI3K-pathway inhibitors in chordoma cell lines whose genomic profiling identified alterations involving PTEN, EGFR, and CDKN2A, while buparlisib significantly inhibited tumor growth in only a subset of the xenograft models tested [26]. Subsequent experiments in p16^INK4A-deficient chordoma models suggested that concomitant PTEN deficiency may provide additional rationale for PI3K- or mTOR-directed treatment strategies [23]. However, neither study established CDKN2A alteration as a predictive biomarker of response to PI3K- or mTOR-pathway inhibition [23,26].

Other chordoma-directed approaches have targeted EGFR, PDGFR, and TBXT/brachyury. Combining EGFR and mTOR inhibition with afatinib and AZD8055 synergistically reduced chordoma cell viability and improved tumor-growth control in preclinical models, although tumor regression was not observed [32]. Pharmacological inhibition of the H3K27 demethylases KDM6A/B reduced TBXT expression and promoted chordoma cell death [33]. Clinically, PDGFR-directed treatment with imatinib has demonstrated predominantly disease-stabilizing activity in advanced PDGFB/PDGFRB-positive chordoma [34]. Importantly, the EGFR/mTOR and TBXT studies did not evaluate or stratify therapeutic response according to CDKN2A or p16^INK4A status, while the imatinib trial selected patients based on PDGFB/PDGFRB expression rather than CDKN2A alteration [32,33,34]. Therefore, the observed therapeutic activity cannot currently be attributed specifically to CDKN2A loss.

Evidence concerning MTAP, SMARCB1/EZH2-directed therapy, and immunotherapeutic strategies remains preliminary in relation to CDKN2A. MTAP loss has been observed in a subset of chordomas and was significantly associated with concurrent loss of p16^INK4A expression [20]. However, no primary chordoma study identified in our targeted search evaluated a PRMT5 or MAT2A inhibitor in an MTAP-selected model or patient cohort. EZH2 inhibition with tazemetostat demonstrated preliminary clinical activity in SMARCB1/INI1-deficient poorly differentiated chordoma, although this represents a distinct molecular subgroup and CDKN2A status was not evaluated [35]. More recently, camrelizumab combined with the antiangiogenic agent apatinib demonstrated clinical activity in refractory chordoma, while exploratory analyses associated CDKN2A copy-number or homozygous deletion with lower response rates and shorter progression-free survival [36]. Because this was a single-arm combination trial, it remains uncertain whether CDKN2A is a treatment-predictive biomarker for PD-1 blockade, antiangiogenic therapy, or their combination.

### 3.7. Clinical Trials

A multi-center phase II clinical trial led by the University Hospital Heidelberg evaluated palbociclib in patients with unresectable or metastatic chordoma [37,38]. The study was a nonrandomized, single-arm, open-label, multicenter phase II trial. Palbociclib was administered at 125 mg once daily on days 1–21 of each 28 day cycle. This trial enrolled patients with p16 loss (confirmed via IHC) or CDKN2A loss (confirmed by genomic analyses) along with simultaneous retained CDK4/6 and RB1 expression. Most patients had previously undergone surgery and radiotherapy, and 75% had received prior systemic therapy. The median number of prior systemic treatment lines was one (range, 0–5). A total of 28 patients were reported in the results, with disease control rate (DCR) at six cycles as the primary endpoint. Three patients experienced prolonged stable disease lasting 19, 27, and 42 months, indicating that a small subset obtained durable disease control despite the absence of objective responses. Disease stability, with no objective responses, was achieved in 11 patients (39%), meeting the primary endpoint. The median progression-free survival was 5.6 months, and the median overall survival was 24.6 months. Treatment-related adverse events occurred in 96.4% of patients, with grade 3 events occurring in 39.2%. Neutropenia was the most frequent grade 3 toxicity, which occurred in 28.5% of patients. The authors hypothesized, based on preclinical results on chordoma cell lines, that the proportion of tumor cells expressing pRB may stratify responder phenotypes. However, they observed no outcome differences by pRB expression. Although the trial met its predefined disease-control endpoint, the absence of objective responses indicates that the clinical activity of palbociclib monotherapy was modest. The lack of association between pRB expression and outcome further suggests that p16 or CDKN2A loss with retained RB1 expression is insufficient as a standalone predictive biomarker. Additional potentially actionable alterations involving PIK3CA, PTEN, MTAP, and MET support the investigation of broader molecular-selection strategies and rational combination therapies.

In addition to the completed palbociclib trial, an ongoing single-arm phase II study sponsored by the Medical College of Wisconsin is evaluating abemaciclib in advanced bone and soft-tissue sarcomas, including chordoma. These were selected for CDK-pathway alterations such as CDKN2A/B copy-number loss or cyclin D-CDK4/6 amplification. Retention of Rb expression was required for eligibility; however, no results have been posted [39]. A separate phase II study, PalboSarc, includes a dedicated chordoma cohort selected for CDKN2A mutation; however, its current status is listed as unknown, and no chordoma-specific results have been reported [40]. Finally, a phase II trial of camrelizumab plus apatinib enrolled patients with refractory chordoma without CDKN2A-based selection. Exploratory analyses associated CDKN2A copy-number or homozygous deletion with poorer prognosis, although the study did not establish CDKN2A as a predictive biomarker for either therapy (Table 3) [36].

## 4. Discussion

Chordoma remains a difficult disease to treat because local control is challenging, recurrence is common, and effective systemic therapies remain limited. In that context, identifying molecular alterations that are both biologically meaningful and therapeutically actionable is critical. The literature reviewed here suggests that CDKN2A occupies an important position at the intersection of chordoma biology, prognosis, and therapeutic development. Across genomic, epigenetic, and protein-expression studies, CDKN2A loss or downstream loss of p16^INK4A emerges as a recurrent event that has been associated, in selected cohorts, with adverse clinicopathologic features and poorer outcomes; however, its independent prognostic value remains inconsistently demonstrated [13,15,17,18,19,20]. More importantly, because CDKN2A inactivation directly disrupts the CDK4/6–Rb cell-cycle checkpoint, it also provides a biologically plausible rationale for targeted therapeutic intervention [5,11,12,13,14,15,16,17,18,19,20,21,22,23,24].

The most consistent finding across the reviewed studies is that deletion of the 9p21 locus, often involving CDKN2A with or without CDKN2B, is among the most recurrent genomic alterations in chordoma [16]. However, the reported prevalence varies substantially across cohorts, ranging from relatively low rates in some skull base series to much higher rates in spinal and sacral tumors [11,12,13,14,15,16,17,18,19,21]. This variability likely reflects several factors, including differences in tumor location, cohort composition, assay, and whether studies evaluated gene deletion, copy number loss, loss of heterozygosity, or p16^INK4A protein expression. These data support the view that chordoma is not genomically uniform across anatomical sites and that the prevalence and clinical significance of CDKN2A alteration may differ between skull base and non-skull base tumors [11,12,13,14,15,16,17,18,19,21].

CDKN2A loss has been associated with features suggestive of more aggressive tumor biology, although its prognostic significance is inconsistent across studies. Multiple investigations reported associations between CDKN2A deletion or p16^INK4A loss to shorter progression-free or disease-free survival, higher Ki-67 indices, recurrent disease, adverse methylation-defined subgroups, or increased rates of progression [13,15,19,20,21,22,23]. Simultaneously, not all studies identified an independent survival association, and some showed significance only in univariate models or failed to demonstrate a direct relationship with overall survival [16,17,20]. These mixed findings indicate that the prognostic significance of CDKN2A/p16 alteration remains uncertain and may depend on anatomical site, assay methodology, cohort composition, co-occurring alterations, and the clinical endpoint evaluated. The studies differed in several important aspects. Assessment methods varied, with some studies evaluating genomic deletion by FISH or sequencing and others relying on immunohistochemistry for p16^INK4A expression. Cohort composition also differed, as certain series were restricted to skull base chordomas, whereas others included spinal and sacral lesions. Finally, the reported endpoints were inconsistent, spanning progression-free survival, disease-free survival, overall survival, and progression status. Collectively, the available evidence supports a hypothesis-generating association between CDKN2A/p16 alteration and adverse tumor behavior, but does not yet establish CDKN2A loss as a consistently independent prognostic biomarker across chordoma populations [12,13,15,16,17,18,19,20,21,22,23]. For example, p16^INK4A loss in Sommer et al. and CDKN2A/B loss in Passeri et al. were associated with adverse outcomes in univariate analyses but did not retain independent significance after multivariable adjustment, whereas other studies demonstrated associations within broader molecular risk groups or with progression without establishing an independent CDKN2A-specific effect. In contrast, 9p21 deletion burden in skull-base chordoma has shown stronger prognostic value in multivariable risk modeling [13,20].

An important nuance in this literature is that p16^INK4A loss cannot be explained by homozygous CDKN2A deletion alone. Several studies demonstrated discordance between genomic status and protein expression, indicating that additional mechanisms contribute to functional pathway inactivation [11,12]. Promoter methylation appears to account for only a minority of such cases, suggesting that other mechanisms, including focal mutation, post-transcriptional regulation, or translational repression, may also contribute [7,11,12]. This distinction is clinically relevant because p16^INK4A loss may reflect a functional pathway disruption rather than copy-number status alone. It also underscores a broader challenge in biomarker development for chordoma: gene deletion, protein loss, and downstream pathway dependence are closely related, but they are not interchangeable, and future studies will need to define more clearly which of these measures is most predictive of prognosis and therapeutic response [7,11,12,24].

The translational significance of CDKN2A loss becomes most compelling when considered in the context of cell-cycle dependence. Loss of p16^INK4A relieves inhibition of CDK4/6, permits Rb phosphorylation, and promotes unchecked G1–S transition [4,5,6]. Preclinical studies reviewed here support this biologic model, demonstrating that chordoma cell lines and xenografts with CDKN2A loss or p16^INK4A deficiency are often susceptible to CDK4/6 inhibition [23,24,25,26]. To that point, von Witzleben et al. showed that many chordoma tissues exhibited loss of p16 expression while retaining phosphorylated Rb, consistent with continued downstream dependence on the CDK4/6–Rb axis despite upstream loss of cell-cycle inhibition [24]. In parallel, patient-derived chordoma cell lines with CDKN2A loss demonstrated sensitivity to palbociclib and abemaciclib, although the degree of response varied substantially across models [24]. These findings support the concept that CDKN2A loss may define a therapeutically relevant state of cell-cycle vulnerability, but they also make clear that CDKN2A status alone is unlikely to fully explain treatment sensitivity. Importantly, the observed responses were predominantly cytostatic, characterized by cell-cycle arrest and partial growth inhibition rather than substantial apoptosis or tumor regression.

That heterogeneity in therapeutic response likely reflects the broader molecular context in which CDKN2A loss occurs. Several studies suggest that co-alterations involving PTEN, the PI3K/AKT/mTOR pathway, MTAP, or chromatin-remodeling genes such as SMARCB1 may shape both tumor behavior and response to therapy [7,20,23,27,28,29,41]. In this framework, CDKN2A loss may be best understood not as an isolated driver, but as one component of a broader oncogenic program that promotes proliferation while interacting with complementary survival pathways. This may explain why CDK4/6 inhibition alone often suppresses proliferation without fully eliminating tumor growth, whereas combination strategies appear more effective in preclinical models [23,24,25,26]. In particular, the enhanced effects observed with combined palbociclib and mTOR inhibition support a model in which selected dual-pathway strategies may help overcome biologic redundancy and adaptive resistance in a subset of chordomas. However, the absence of synergy with all tested P13K-pathway combinations indicates that such approaches will require molecularly informed selection [23,26,27,28,29].

From a clinical perspective, the available evidence supports two practical conclusions. First, CDKN2A has emerging value as a stratification biomarker, particularly when interpreted within broader molecular risk models rather than in isolation. This is exemplified by studies integrating 9p21 deletion burden with other alterations such as 1p36 loss or PTEN deficiency to refine prognostic classification [22,23]. Second, CDKN2A-deficient chordomas represent a rational population for CDK4/6-targeted therapy, although current evidence suggests that patient selection will likely need to extend beyond simple deletion status alone. Features such as p16^INK4A loss, retained Rb pathway signaling, and relevant co-alterations in PI3K/mTOR-associated pathways may ultimately prove more useful in identifying responders [23,24,37,38]. The recent phase II palbociclib trial demonstrates the clinical feasibility of targeting this pathway; however, the absence of objective responses and the modest disease control observed underscore the translational gap between preclinical pathway sensitivity and meaningful clinical benefit, as well as the limitations of monotherapy. Thus, the available clinical evidence remains limited and illustrates several challenges in translating preclinical CDK4/6 sensitivity into clinical benefit. Although the completed palbociclib trial met its predefined disease-control endpoint, no objective responses were observed, suggesting predominantly cytostatic rather than tumor-regressive activity [37,38]. Furthermore, patient-selection strategies also differ across studies, ranging from p16^INK4A or CDKN2A loss with retained RB1 expression to broader CDK-pathway alterations [37,39,40]. Small chordoma-specific cohorts, heterogeneous biomarker definitions, the absence of randomized control groups, and the difficulty of interpreting stable disease in a frequently slow-growing tumor further limit cross-trial comparison and biomarker validation [37,39,40]. Consequently, neither CDKN2A alteration nor retained Rb expression has yet been established as a reliable predictor of clinical benefit from CDK4/6 inhibition.

### 4.1. Proposed Biomarker-Informed Framework for CDK4/6-Directed Therapy

Since the available evidence indicates that CDKN2A copy-number status is unlikely to be sufficient for selecting patients with chordoma for CDK4/6-targeted therapy, a practical selection framework should therefore begin by assessing CDKN2A copy number and p16^INK4A expression together. Tumors with confirmed p16^INK4A loss should then be evaluated for retained RB1/Rb and CDK4/6 expression, as preservation of this downstream pathway provides the clearest biological rationale for CDK4/6 inhibition.

Accordingly, tumors with p16^INK4A loss and retained RB1/Rb expression may represent the most appropriate candidates for CDK4/6 inhibitor therapy within a clinical trial. However, this profile should not be considered predictive of response. The phase II palbociclib trial enrolled patients using similar criteria but demonstrated only modest disease control, no objective responses, and no association between pRB expression and outcome [37,38]. Thus, p16 loss and RB preservation identify biological plausibility rather than a validated responder population. PTEN and PI3K/AKT/mTOR pathway status may provide an additional level of stratification.

In tumors with p16^INK4A loss, retained RB1/Rb, and concurrent PTEN deficiency, combined CDK4/6 and mTORC1 inhibition may be appropriate for investigation based on the enhanced preclinical activity of palbociclib plus rapamycin [25]. This rationale should not be generalized to all PI3K-pathway combinations, because palbociclib plus buparlisib did not demonstrate synergy. MTAP and SMARCB1 alterations may identify separate therapeutic vulnerabilities but have not been established as predictors of CDK4/6 inhibitor response. Therefore, the framework summarized in Table 4 should be considered hypothesis-generating and requires prospective validation before clinical implementation.

**Table 4:** Molecularly targeted clinical trials relevant to chordoma.

Trial/Reference	Agent/Strategy	Phase/Design	Chordoma Population/Biomarker Selection	Status/Key Findings	Relationship to CDKN2A
NCT03110744 [37,38]	Palbociclib CDK4/6 inhibition	Phase II; nonrandomized, single-arm, open-label, multicenter	Unresectable or metastatic chordoma with p16 loss by IHC or CDKN2A loss by genomic analysis; retained CDK4/6 and RB1 expression required.	Completed. n = 28; DCR at 6 cycles 39% (11/28), all stable disease. Median PFS 5.6 months. Median OS 24.6 months. Three patients had prolonged stable disease of 19, 27, and 42 months.	Directly CDKN2A/p16-selected. Clinical activity was modest, and pRB expression was not associated with outcome; CDKN2A/p16 status with retained RB1 was not established as a standalone predictive biomarker.
NCT04040205 [39]	Abemaciclib CDK4/6 inhibition	Phase II; single-arm basket study	Advanced bone and soft-tissue sarcomas, including chordoma, selected for CDK-pathway alterations such as CDKN2A/B copy-number loss or cyclin D–CDK4/6 amplification; retained Rb expression required.	Ongoing; no results posted.	Directly relevant. Tests a broader biomarker-selection strategy incorporating CDKN2A/B or other CDK-pathway alterations together with Rb retention.
NCT03242382 (PalboSarc) [40]	Palbociclib CDK4/6 inhibition	Phase II	Includes a dedicated chordoma cohort selected for CDKN2A mutation.	Current status listed as unknown; no chordoma-specific results reported.	Directly relevant, but efficacy in the CDKN2A-selected chordoma cohort remains undetermined.
Yang et al. [36]	Camrelizumab + apatinib PD-1 inhibition + antiangiogenic therapy	Phase II; refractory chordoma	Refractory chordoma without CDKN2A-based enrollment selection.	Clinical activity observed. Exploratory molecular analyses associated CDKN2A copy-number or homozygous deletion with poorer outcomes.	Exploratory only. CDKN2A alteration was associated with prognosis but was not established as a predictive biomarker for treatment response.

### 4.2. Future Research Priorities

Considering the reported frequencies of CDKN2A alteration vary across skull-base, spinal, and sacral chordomas, which suggest that anatomical location influences both molecular prevalence and clinical significance, future cohorts should enroll sufficient numbers from each anatomical site and analyze these groups separately rather than combining them into a single heterogenous population [11,13,14,15,17]. Standardized assessment of CDKN2A copy number, p16^INK4A expression, and clinical outcomes would also improve comparison across studies, particularly because genomic deletion and protein loss are related but not interchangeable [11,12].

Biomarker-guided patient stratification should extend beyond CDKN2A status alone. Future studies should evaluate whether composite molecular profiles incorporating p16^INK4A expression, RB1/Rb preservation, and PTEN/PI3K/AKT/mTOR pathway alterations provide greater prognostic or predictive value than any individual marker [23,24,37]. Molecular risk models integrating CDKN2A-region alterations with additional genomic features may similarly improve prognostic classification and support a more precision-oriented approach to trial enrollment [21,23]. These biomarker panels should be evaluated prospectively using consistent assays and multivariable analyses to determine whether they independently predict treatment response or clinical outcome.

Combination therapies represent another important priority. Preclinical findings support further investigation of CDK4/6 inhibition combined with complementary pathway-directed agents, particularly mTOR inhibition in p16^INK4A- and PTEN-deficient models [23]. However, because not all PI3K- or mTOR-directed combinations have demonstrated equivalent activity, future trials should select combinations according to the molecular profile of the tumor rather than pathway alterations alone [23,26]. Paired pretreatment and post-progression specimens should also be incorporated where it is feasible to identify acquired resistance mechanisms and distinguish treatment-emergent molecular changes from pre-existing tumor heterogeneity [13,42]. Collectively, these approaches may help translate CDKN2A-associated biology into more reliable prognostic models, biomarker-enriched trials, and rational combination strategies.

### 4.3. Limitations

There are several limitations that must be considered in this review. The studies reviewed here are heterogeneous in methodology, including use of immunohistochemistry, FISH, methylation profiling, targeted sequencing, whole-exome sequencing, and whole-genome sequencing, making direct comparison difficult. Many cohorts are also small, retrospective, and enriched for anatomical subtypes, which limits both generalizability and statistical power. In addition, much of the therapeutic evidence remains preclinical, and the relationship between genomic CDKN2A loss, functional p16^INK4A deficiency, and true clinical drug sensitivity has not yet been standardized. Finally, demographic correlates of CDKN2A alteration remain poorly characterized: age- and sex-based differences were not directly assessed in the studies reviewed, and the predominance of single-institution and regionally restricted cohorts raises the possibility of unexamined population-level variation in CDKN2A and related genomic alterations. Clarifying how patient demographics relate to these genomic changes represents an important direction for future study. These limitations highlight the need for prospective, biomarker-enriched studies using consistent molecular definitions and clinically meaningful endpoints.

## 5. Conclusions

Overall, the literature supports a model in which CDKN2A loss is not simply a recurrent genomic event in chordoma, but a biologically and clinically important alteration that helps define a more proliferative and potentially targetable disease state. The next challenge is not merely to document its presence, but to incorporate it into a more precise therapeutic framework. For chordoma, that will likely require biomarker-guided combination strategies that integrate CDKN2A status with pathway context, rather than reliance on single-marker or single-agent approaches alone.

## Figures and Tables

**Table 2:** Summary of therapeutic agents, respective chordoma genetic profiles tested on, and efficacy.

Drug Tested	Cell Line/Xenograft + Anatomical Origin	Model/Assay Type	Genetic/Biomarker Profile	Efficacy Results	Mechanism of Action	Clinical/Translational Interpretation
Palbociclib	Various chordoma cell lines; Sacralcoccygeal chordoma; mix of primary and recurrent chordoma	In vitro cell-viability/proliferation assays; FISH and expression profiling	50–86% CDKN2A loss; CDK4/6 and Rb expression retained; all cell lines express brachyury	Up to 18.5% reduction in proliferation; G1-phase arrest; no cleaved PARP or caspase-3	Selective CDK4/6 inhibition; restrains G1–S progression	Supports pathway activity but not tumor-cell eradication; response varies across models
Palbociclib	Three patient-derived xenografts; Sacral primary; previously irradiated skull-base tumor; skull-base primary	In vivo patient-derived xenograft treatment study	Two xenografts with CDKN2A/B deletion; one PBRM1-mutant model without CDKN2A/B deletion	CDKN2A/B-deleted models: 58–62% TGI; PBRM1-mutant model: 12% TGI	Selective CDK4/6 inhibition	Greater activity in the deleted models, but only three xenografts were studied
Palbociclib	UM-Ch1 and UM-Ch2; Unspecified	In vitro dose–response assay	UM-Ch1: no CDKN2A alteration; UM-Ch2: homozygous CDKN2A deletion	UM-Ch2 IC50 = 7.4 µM; UM-Ch1 resistant at the tested range	Selective CDK4/6 inhibition	Supports a possible relationship between CDKN2A deletion and sensitivity, but the high IC50 limits direct clinical extrapolation
Flavopiridol	UM-Ch1 and UM-Ch2; Unspecified	In vitro dose–response assay	UM-Ch1: no CDKN2A alteration; UM-Ch2: homozygous CDKN2A deletion	Lower IC50 than palbociclib; IC50 = 0.3 µM in UM-Ch2	Broad-spectrum CDK inhibition (CDK1, CDK2, CDK4, and CDK6)	Suggests broader CDK inhibition may overcome some pathway redundancy, but clinical relevance is unproven
Abemaciclib	U-CH2, U-CH3, U-CH6, U-CH7, and U-CH11; Sacralcoccygeal chordoma; mix of primary and recurrent chordoma	In vitro cell-viability/dose–response assay	Variable CDKN2A loss and pathway context	IC50 = 50–100 nM in U-CH2, U-CH3, U-CH6, and U-CH7; 300–400 nM in U-CH11	Selective CDK4/6 inhibition	Substantial between-model variability indicates that CDKN2A status alone is unlikely to predict response
Buparlisib + Rapamycin	p16- and PTEN-deficient cell lines; Unspeciffied	In vitro combination-treatment assay	p16 and PTEN deficiencies	G1-phase arrest and increased cleaved PARP and caspase-3 reported with combination treatment	PI3K and mTORC1 inhibition	Supports rational pathway combinations in selected molecular contexts; verify which agents were combined with palbociclib in the source study

**Table 3:** Proposed biomarker-informed framework for CDK4/6-directed therapy in chordoma.

Biomarker Profile	Biological Interpretation	Potential Therapeutic Approach	Evidence Level and Limitations
CDKN2A loss alone	Genomic alteration without confirmation of p16INK4A deficiency or functional dependence on the CDK4/6–RB pathway.	Insufficient as a standalone treatment-selection criterion. Complementary assessment of p16INK4A expression and functional RB-pathway integrity should be considered.	CDKN2A copy-number status may be discordant with p16INK4A protein expression and therapeutic response. No prospectively validated selection strategy currently supports CDKN2A loss alone.
p16INK4A loss + intact functional RB signaling	Loss of upstream CDK4/6 inhibition with preservation of the downstream RB target provides the strongest biological rationale for CDK4/6 blockade.	Consider CDK4/6 inhibition, preferably within a biomarker-enriched clinical trial.	Preclinical rationale and clinical feasibility have been demonstrated; however, phase II activity was modest, no objective responses were observed, and pRB expression did not predict outcome.
p16INK4A loss + intact RB + PTEN loss or PI3K/AKT/mTOR activation	Potential CDK4/6 dependence accompanied by parallel prosurvival signaling that may limit the activity of CDK4/6 monotherapy.	Investigate rational combinations of CDK4/6 inhibition with mTOR- or PI3K-pathway agents in molecularly selected trials.	Evidence is predominantly preclinical, and not all tested PI3K-pathway combinations have demonstrated synergy. Optimal partner, sequencing, and selection criteria remain undefined.
RB1 loss or functional RB inactivation	Loss of the downstream target required for canonical CDK4/6-inhibitor activity may permit CDK4/6-independent cell-cycle progression.	CDK4/6 inhibition is less biologically compelling; alternative pathway-directed strategies should be prioritized for investigation.	Mechanistic support is strong in other malignancies, but chordoma-specific prospective validation is limited. Functional RB assessment is not yet standardized.
MTAP or SMARCB1 co-alteration	May identify separate or complementary molecular vulnerabilities rather than direct determinants of CDK4/6 dependence.	Consider alternative or combination strategies within molecularly matched clinical trials.	These alterations are not established predictors of CDK4/6-inhibitor response. Their therapeutic relevance should be interpreted independently and validated prospectively.

## Data Availability

No new data were created or analyzed in this study. Data sharing is not applicable to this article.
